# Perceptions and Experiences of Women Participating in a Digital Technology–Based Physical Activity Intervention (the mPED Trial): Qualitative Study

**DOI:** 10.2196/13570

**Published:** 2019-12-20

**Authors:** Teri Lindgren, Julie Hooper, Yoshimi Fukuoka

**Affiliations:** 1 School of Nursing University of California, San Francisco San Francisco, CA United States; 2 Gillings School of Global Public Health University of North Carolina Chapel Hill, NC United States; 3 Physiological Nursing Univesity of California, San Francisco San Francisco, CA United States

**Keywords:** mHealth, randomized controlled trial, behavior, interview, physical activity, maintenance, women, motivation, accountability

## Abstract

**Background:**

Despite the benefits of regular physical activity, women in every age group have lower activity levels than men, and few women meet the recommended levels of physical activity. Digital technologies have been useful in increasing physical activity during the course of an interventional study. However, sustaining that activity once the clinical trial was complete was a major challenge.

**Objective:**

This study aimed to describe the experiences and perspectives of physically inactive women who completed the mobile phone–based physical activity education (mPED), a randomized controlled trial, at 12 months.

**Methods:**

Of 210 women who were enrolled in the mPED trial, 203 completed a 12-month open-ended exit interview and survey through phone. The participants were asked about their physical activity levels; their digital technology use; what they learned from, liked, and would change about the trial; their motivations to keep active post-trial; and their advice for other women. Interviews were transcribed verbatim and thematically analyzed using the brief survey qualitative description. Descriptive statistics were used to describe the survey data with the significance level set at *P*<.05.

**Results:**

In the 12-month survey, a greater proportion of the participants in the intervention group, compared with the control group, reported that they regularly wore a pedometer or physical activity tracker (49.3%, 66/143 vs 26.1%, 18/69; *P*=.002) and engaged in brisk walking (54.5%, 73/134 vs 30.4%, 21/69; *P*=.001). The experiences and perceptions of physical activity of physically inactive women over time were embedded in a complex interplay of internal and external factors. A total of 6 interactive themes emerged as critical in supporting continued engagement in physical activity postintervention: tracking, technology versus personal touch, accountability, resources and environment, motivation, and habit formation. Technology allowed for self-tracking, which supported internal accountability. However, tracking by another person (personal touch) was needed for external accountability. Resources and environment underpinned the relationship among the themes of tracking, technology versus personal touch, accountability, motivation, and habit formation.

**Conclusions:**

Future research is needed to identify the best ways to harness this dynamic process in promoting and sustaining physical activity among inactive women. Digital technology is evolving at an exponential rate and provides new opportunities to transform research into new approaches to promote physical activity.

**Trial Registration:**

ClinicalTrials.gov NCT01280812; https://clinicaltrials.gov/ct2/show/NCT01280812

**International Registered Report Identifier (IRRID):**

RR2-10.1186/1471-2485-11-933

## Introduction

Despite the numerous benefits of physical activity for women, few women meet the recommended levels of physical activity [1,2]. In fact, women in every age group self-report lower activity levels than men [3]. Research has demonstrated that regular physical activity is associated with a reduced risk for chronic illnesses, such as type 2 diabetes, hypertension, and some cancers [3-8]. The Physical Activity Guidelines for Americans—2nd edition recommends that adults should engage in at least 150 to 300 min a week of moderate-intensity activity or 75 to 150 min a week of vigorous-intensity aerobic activity [9]. Yet, self-reported surveys administered nationally show that only 49% of adults in the United States met these recommended minimum activity levels [10]. Given that comparisons between self-report and accelerometer data demonstrate that people tend to overestimate their physical activity, the actual percentage of adults meeting minimum levels of activity is likely to be lower [11].

Use of digital technologies such as mobile phone, apps, and activity trackers to encourage physical activity has gained popularity. The prevalence of mobile phone ownership has significantly increased, reaching 77% in the United States in 2015 [12]. Similarly, the availability of activity trackers or accelerometers that connect with mobile phone health apps has also grown. According to a 2015 Pew Research Center survey, more than half of mobile phone users had downloaded a health app, with fitness and nutrition apps being the most common categories of health apps downloaded [13].

Research has shown that mobile phone apps or activity trackers or accelerometers seem to improve physical activity and reduce sedentary behaviors, at least in the short term [14-17]; however, few clinical trials using digital technology–based interventions to increase physical activity have examined the sustainability of these interventions [18-21]. To help address this knowledge gap, we recently completed the mobile phone–based physical activity education program (mPED) study, a randomized controlled trial (RCT) designed to examine the efficacy of a 3-month mobile app and accelerometer-based physical activity intervention and a 6-month maintenance intervention for physically inactive women (see the study design in Multimedia Appendix 1). The main outcomes of the trial have been previously published [22]. In sum, subjects in the intervention (regular and plus) groups, compared with the control group, substantially increased their accelerometer-measured daily steps by an average net difference of 2060 steps per day at 3 months (95% CI 1296-2825) and 1360 steps per day at 9 months (95% CI 694-2026) and a net difference of moderate-to-vigorous physical activity of 18.2 min per day at 3 months (95% CI 10.9-25.4) and 8.4 min per day at 9 months (95% CI 2.0-14.9). In the plus group, who kept the trial app and accelerometer for an additional 6 months, there were no additional improvements in physical activity compared with the regular group who kept only the accelerometer [22]. Within the control group, the participants significantly increased their physical activity levels (approximately 1000 steps per day) from baseline through 9 months. To explore the experience of continuing physical activity once the study digital technologies were removed from participants, we conducted a telephone interview at 12 months with women who completed the mPED trial to see if their activity level changed after the final 9-month research office visit and the reasons why participants did or did not continue to engage in physical activity. Therefore, the aim of this paper was to explore physically inactive women’s experiences and perspectives on participating in and continuing to engage in physical activity after the 9-month visit.

## Methods

### Study Design and Sample

We used qualitative description to elicit participants’ experiences and perspectives of their engagement during the mPED trial and after the 9-month final office visit [23,24]. Participants were interviewed through phone using a quantitative survey and qualitative interview (open-ended questions) at 12 months. The study protocol was approved by the University of California, San Francisco, Committee on Human Research and the mPED Data and Safety Monitoring Board. Detailed descriptions of the study design and outcomes have been previously published [15,22,25,26]. In short, physically inactive women aged 25 to 69 years were recruited from the San Francisco Bay Area between May 2011 and April 2014. The Social Cognitive Theory [27] and the Stages of Change Model (SCM) [28] were used to guide the design of the trial, and the SCM was also used to identify participants who were in the *contemplation* or *preparation* stages of behavior change (ie, an appropriate target study population for the intervention). During the telephone screening, research staff assessed participants’ behavior change readiness (*contemplation or preparation*).

In brief, the mPED trial was an unblinded, parallel RCT conducted with 3 groups (control, regular, and plus groups; see Multimedia Appendix 1). The trial consisted of a 3-week run-in period, a 3-month intervention period, and a 6-month maintenance period. The control group was asked to use an Omron Active Style Pro HJA-350IT (Omron Healthcare) accelerometer to record and store physical activity every day for the entire 9-month study period but did not receive any physical activity intervention. In contrast, the regular and plus groups received the identical physical activity intervention, consisting of an accelerometer, brief in-person counseling sessions, and the mPED trial app for the first 3 months. The mPED trial app developed by the research team has 2 main functions: (1) a daily message or video clip and (2) a daily diary. The trial app provided each participant’s weekly daily step goals, which were set to increase at a 20% rate from each study participant’s average baseline daily steps. Once daily step goals reached 10,000 steps, the study participant was asked to maintain at least 10,000 steps per day, 7 days a week during the remaining study period. Personalized automated feedback was provided daily via the mPED trial app. In addition, in the 6-month maintenance period, the plus group kept using both the mPED trial app and accelerometer, whereas the regular group kept using only the accelerometer. In this trial, the research team used the term *pedometer*, instead of accelerometer, to the study participants.

### Procedures for the 12-Month Data Collection

At the 9-month visit, all participants returned all research equipment, including accelerometers and study mobile phones (if any) to the research office. If the study app was installed on their mobile phones, the research staff removed the study app. Participants were encouraged to purchase an activity tracker, if they reported that they did not have one, using the US $40 compensation for their time. At the end of the 9-month visit, all participants scheduled a 12-month follow-up telephone interview and then received a text, an email, or a telephone call to confirm their 12-month telephone interview appointment. The 12-month interview consists of 2 parts: (1) a survey and (2) a semistructured interview using open-ended questions (see Multimedia Appendix 2). Owing to the need to have a sufficient sample for the survey, all 203 participants were interviewed quantitatively and qualitatively. The interviews were conducted over the telephone by research assistants trained in both interviewing techniques [29]. Interviews were digitally recorded and transcribed verbatim by a professional transcriptionist. The average length of the semistructured interviews was 18 (SD 6) min, ranging from 7 to 41 min.

### Analytic Strategy

Transcribed interview data were imported into ATLAS.ti 8.0 to assist in qualitative data analysis. Alphanumeric identifiers were used to ensure participant confidentiality, and audio files were kept on a secure device in a locked drawer in the research office. A total of 3 researchers reviewed 10 transcripts to inductively develop the initial codebook based on the answers provided to the research questions. Overall, 10 interviews were chosen because some of the interviews were relatively short, and the normally recommended number of 3 interviews was too small to sufficiently capture emerging codes [30]. After reviewing and comparing codes for how well the codes were capturing the perspectives of the participants, agreement on the coding scheme was achieved. Overall, 15% (30/203) of the transcripts were doubled coded, with 90% intercoder agreement. One researcher then coded the rest of the interviews for consistency. The 3 investigators reviewed the coding weekly within and across all interviews and discussed emerging commonalities. As the trial was effective in improving physical activity outcomes [22], we presented the qualitative interview data between the control versus intervention (regular and plus) groups. Constant and collaborative reviewing of data led to collapsing and grouping of codes into broader categories reflective of emerging themes that were evident across all 3 research groups [30]. Although theory development was not a goal, we sought more conceptual parsimony in the themes than in the descriptions [31]. Further examination, merging, connecting, and refining of codes within themes allowed for clarification of meaning and identification of patterns and relationships among the themes. Quotes associated with collapsed categories were then examined collectively by the research team to clarify broader themes across all groups (see Multimedia Appendix 2) and determine which best described the final themes. For the survey data, descriptive statistics were used with the significance level set at *P*<.05. As there was no statistical difference in the physical activity outcomes between the 2 intervention (regular and plus) groups [22], the 2 intervention groups were combined in this paper.

## Results

### Quantitative Findings

Overall, 96.7% (203/210) of the mPED trial participants completed the 12-month phone interview. The baseline characteristics in the sample of 203 participants did not differ from the 7 nonparticipants (*P*>.05). Baseline demographics are presented in Table 1. Mean participant age was 52.6 (SD 11.0) years, 56.7% (115/203) self-identified as non-Hispanic white, 74.4% (151/203) had a full- or part-time job, and 74.9% (152/203) completed 4 years of college. There was no difference in baseline characteristics between the control and intervention groups (*P*>.05).

At 12 months, 67.2 % (90/134) of participants in the intervention group and 51.5% (35/69) of participants in the control group reported that they owned a pedometer or activity tracker (*P*=.03), whereas 49.3% (66/134) of participants in the intervention group and 26% (18/69) of participants in the control group reported that they regularly wore the pedometer or activity tracker (*P*=.002; Table 2). In response to the question “Has your physical activity been more, less, or about the same compared with the first 9 months of the study?,” a significantly higher proportion of participants in the control group, compared with the intervention group, reported engaging in more physical activity from 9 to 12 months (*P*=.001). However, a greater proportion of participants in the intervention group engaged in more brisk walking compared with the control group (*P*=.001).

**Table 1:** Baseline demographics of mobile phone–based physical activity education participants who completed the 12-month interview.

Characteristics		Total (N=203)	Control (n=69)	Intervention (regular and plus groups; n=134)	*P* value
Age (years), mean (SD)		52.6 (11.0)	52.0 (9.9)	52.8 (11.5)	.66
**Race and ethnicity, n (%)**					.46
	African American	16 (7.9)	3 (4)	8 (6.0)	
	Hispanic or Latino	11 (5.4)	3 (4)	8 (6.0)	
	Asian	41 (20.2)	13 (18)	28 (20.9)	
	White (non-Hispanic)	115 (56.7)	36 (52)	79 (59.0)	
	More than 1 race	20 (9.9)	9 (13)	11 (8.2)	
**Education, n (%)**					.14
	Completed high school or some college coursework	51 (25.1)	23 (33)	28 (20.9)	
	Completed college (4 years)	83 (40.9)	24 (34)	59 (44.0)	
	Completed graduate school	69 (34.0)	22 (31)	47 (35.1)	
**Annual household income (US $; before tax), n (%)**					.66
	<40,000	31 (15.3)	13 (18)	18 (13.4)	
	40,001-75,000	49 (24.1)	14 (20)	35 (26.1)	
	>75,000	107 (52.7)	36 (52)	71 (53.0)	
	Decline to state or do not know	16 (7.9)	6 (8)	10 (7.5)	
**Marital status, n (%)**					.24
	Never married, divorced, or widowed	97 (47.8)	29 (42)	68 (50.7)	
	Currently married or cohabitating	106 (52.2)	40 (58)	66 (49.3)	
**Employment, n (%)**					.14
	Employed for pay (full or part time)	151 (74.4)	47 (68)	104 (77.6)	
	Retried or unemployed or homemaker	52 (25.6)	22 (31)	30 (22.4)	
**Living with a child (children), n (%)**					.17
	Yes	50 (24.6)	21 (30)	29 (21.6)	
Body mass index (kg/m^2^), mean (SD)		29.9 (6.2)	30.4 (5)	29.6 (6.2)	.46

**Table 2:** Comparison of app and pedometer use and self-reported physical activity between control and intervention groups.

Survey questions		Overall (N=203), n (%)	Control (n=69), n (%)	Intervention (regular and plus; n=134), n (%)	Overall *P* value
Do you currently have a health-related mobile application? (yes)		84 (42.0)	29 (42)	55 (41.7)	.89
Do you currently wear a pedometer? (yes)		84 (41.4)	18 (26)	66 (49.3)	.002
Do you have your own pedometer? (yes)^a^		125 (61.9)	35 (51)	90 (67.2)	.03
**Reasons for not purchasing a pedometer after the study visit^b^**		n=77	n=33	n=44	N/A^c^
	Still planning to purchase or keep looking	31 (40)	13 (39)	18 (53)	N/A
	Too expensive or financial difficulty	17 (22)	2 (6)	15 (44)	N/A
	Use app or phone or be able to estimate steps	9 (12)	4 (12)	5 (15)	N/A
	Do not help or do not like	8 (10)	6 (18)	2 (6)	N/A
	Technology challenging or not accurate	6 (8)	4 (12)	2 (6)	N/A
	Has one somewhere or has not set up	6 (8)	5 (15)	1 (3)	N/A
	Other	6 (8)	2 (6)	4 (12)	N/A
**Since your 9-month visit, what types of exercise have you engaged in to be physically active? (multiple choice question)^d^**		n=203	n=69	n=134	N/A
	Walking	126 (62.1)	49 (71)	77 (57.5)	.06
	Brisk walking	94 (46.3)	21 (30)	73 (54.5)	.001
	Yoga	20 (9.9)	3 (4)	17 (12.7)	.06
	Hiking	15 (7.4)	5 (7)	10 (7.5)	.96
	Gardening or yard work	16 (7.9)	7 (10)	9 (6.7)	.39
	Cycling	19 (9.4)	7 (10)	12 (9.0)	.78
	Other	110 (54.2)	39 (56)	71 (53.0)	.77
**Since your 9-month study visit, has your physical activity been more, less, or about the same (compared with the first 9 months of the study)?^e^**		n=203	n=69	n=134	N/A
	About the same	66 (32.5)	27 (39)	39 (29.1)	.001
	More	64 (31.5)	29 (42)	35 (26.1)	.001
	Less	73 (36.0)	13 (18)	60 (44.8)	.001
**Top 3 reasons for being less active after the 9-month visit (multiple choice question)^f^**		n=73	n=13	n=60	N/A
	Study ended	20 (28)	0 (0)	20 (33)	N/A
	Lack of time	20 (28)	4 (31)	16 (27)	N/A
	Did not have a pedometer	12 (16)	2 (15)	10 (17)	N/A

^a^Missing 1 participant.

^b^Total N=77, control n=33, intervention n=34.

^c^N/A: not applicable.

^d^N=203 but some subjects answered more than once.

^e^N=203.

^f^Total N=73, control n=13, intervention n=60.

### Qualitative Findings

Overall, participants, regardless of group, appeared to like participating in the trial itself and enjoyed interacting with the research team. Although not all aspects of the study program were addressed in the 12-month interview, participants talked about their experiences with these digital technologies (pedometers and the mobile phone app), increasing their physical activity and challenges in maintaining physical activity. A total of 6 conceptual themes, such as tracking, technology versus personal touch, accountability, environment and resources, motivation, and habit formation, emerged from the data. Multimedia Appendix 2 shows commonalities and small differences between the 2 groups in their experiences of participating in the study and perspectives on maintaining physical activity and motivation poststudy. These themes connect with the survey data reported above.

### Tracking

Participants in all groups talked a lot about the importance of tracking or keeping track of one’s activity to remain motivated:

There is something about keeping track because it is very difficult to discipline yourself, the whole thing is to get to a new habit.

ID 1060, control, age 50 years

Wearing a pedometer was seen as the primary method for tracking by both control and intervention groups as it allowed for one to “check how many steps I walked in one day” [ID 1003, plus, age 33 years].

Two subthemes emerged from participants discussion of tracking: self-awareness (internal tracking) and tracking by others (external tracking).

#### Self-Awareness

Knowing how many steps the participant had taken was also seen as a mechanism of self-awareness as 1 woman in the control group clearly articulated:

I learned how physically inactive I was prior to the study (And how did you learn that?) Just by tracking those steps and seeing...I work remotely so when you’re at home and you are doing 1000 steps a day, that sounds like a lot, you know. You don’t know. And then as time progresses you realize that you didn’t even go to the driveway and back for a thousand steps, that it’s just barely nothing. I just think I was more conscious of being physical active, more physically active...because I had the pedometer on, and it kept me conscious of what I was doing.

ID 1238, control, age 57 years

For some, having a pedometer provided the needed tracking information as it alerted them to the number of steps they had taken that day so that if they had missed the mark (or the expected goal) they could work to increase their steps before bedtime:

In the evening when I text that I haven’t been doing much activity or anything, I will walk until I hit a certain goal or until I am tired and then I will stop and go to bed.

ID 1063, control, age 65 years

Others looked at the pedometer’s tracking ability to get a sense of how well they have done over the course of a week rather than a daily reminder:

I would like the pedometer to keep track of my activity. The daily tracking was boring. What I like about the pedometer is that I could see my previous activity. That I like. So, I know if it is a good week or a bad week and then I will make up the difference in the following week.

ID 1083, plus, age 44 years

However, for some participants, self-awareness and pedometer tracking were not enough to maintain their increased level of physical activity, and they desired external tracking.

#### Tracking by Others

Additional tracking was needed by some participants to encourage them to keep up their physical activity. Although the intervention group discussed this theme more than the control group, participants from both groups talked about the importance of tracking by someone else. This tracking came through having to *report in* to the research team the number of steps per day through either the download of pedometer data (control group) or the research designed app (intervention group). A woman in the control group stated the following:

I like reporting into you. I like reporting my success. It was validation. I like getting validated...I felt value. And I also got validated that each time I was measured and weighed that I was a success.

ID 1291, control, age 59 years

An intervention participant remarked that she “actually liked having to report my steps every day on the phone. I would have liked doing that every day” (ID 1078, regular, age 68 years).

Intervention participants received feedback after reporting in their steps via the phone app. Feedback included praise for meeting their step goals or encouragement to do better in the coming days. One participant noted:

[I realized] how much I needed someone monitoring what I am doing. How, honestly, I could see the difference when I went from being in the group where you reported every night to being in the group that was set free and you only recorded when you went to the soft tablet. That really did make a difference, the daily check-ins, as intrusive as it seems, they were really a factor [in keeping me active].

ID 1113, regular, age 55 years

Tracking was closely linked to participants’ discussion about technology versus personal touch. Pedometers were mechanisms of tracking, and reporting one’s steps required engaging with a human researcher, both of which were important components of tracking.

### Technology Versus Personal Touch

Both control and intervention groups spoke about the importance of technology and personal touch in engaging in physical activity. What was evident in the data was that some embraced the technology. As 1 control participant advised others:

Get a pedometer and, you know, get one with an application attached to it. That you know, it does encourage you to get the steps, and get some physical activity.

ID 1166, control, age 60 years

Others are more technology adverse, as 1 intervention participant stated:

The cell phone was especially bad for me because I turned into one of those people that’s always looking at their phones...The phone made me somewhat antisocial and dehumanized me.

ID 1013, plus, age 66 years

Regardless of their attitude toward technology, 41% (28/69) of the control group stated in their interviews that they liked the pedometer, and 39% (52/134) of the intervention group mentioned that they liked the pedometer and the app, finding them useful in helping them to keep walking. Women in the control group spoke exclusively about the pedometer as that was the only technology they received. Furthermore, 1 control participant statement reflected many others:

I liked having the pedometer. Being able to see even the little bit of activity, like oh walking to the printer or walking across the street to grab lunch, it all adds up.

ID 1182, control, age 39 years

Some women from both groups complained about the size or bulkiness of the pedometer, whereas others found having to wear it daily irritating:

I don’t like wearing a pedometer...it was just like, kind of a pain in the butt, kind of thing. It was just annoying, like, putting it on every day and that kind of thing.

ID 1191, control, age 39

Although the intervention group demonstrated similar perspectives about the pedometer, they also discussed the app provided by the study. As 1 participant noted, the app had both positive and negative aspects:

The app—there were some days that it felt like a really harsh ... supervisor, you know, like, it’d be 8:00pm and I’d think “Oh, I gotta enter that information! And I haven’t done my exercises yet.” I’d feel guilty. But I don’t see that as a bad thing. You know, somebody’s gotta be the task master, ya know, and that helped me be my own.

ID 1245, regular, age 49 years

The app provided external tracking (having to check-in via the app), feedback, and encouragement, which work well for the majority of study participants. Although the control group spoke about monitoring their steps in general, only the intervention group discussed reaching their step goals. Yet, the pedometer (especially for the control group) and the app were not perceived as only technology. There was also a person (a research team member) on the other end monitoring their steps. Women from both groups explicitly stated that they liked or needed to be able to talk to someone, to reach out to someone who was tracking their steps and to whom they would be accountable, which is why this study design worked for them. Although the control group only met with research team members periodically to download their pedometer readings, be measured, and complete survey tools, they also talked about the importance of the personal interactions in keeping them active. As 1 person noted:

I actually liked the check in, coming in and interacting with you. That was encouraging.

ID 1039, control, age 61 years

Discussions about tracking via technology or personal touch reflect the importance of accountability in both initiating physical activity within the study and maintaining it poststudy.

### Accountability

Participants from both groups used the word *accountability* or *being accountable* frequently. *Accountability* involved *having responsibility for* setting a goal and then being held accountable for reaching it. The study itself had built-in accountability, but it varied by group. The control group talked about being held accountable to the research team when they had to come in every study visit and download pedometer data, be measured, and complete questionnaires:

I liked that I could discuss with you and others that worked there and discussed these issues, for the support, measurement itself, and ask questions that I had about this issue. It created some motivation for me.

ID 1077, control, age 59 years

On the contrary, the intervention group talked about the accountability established through reporting daily steps via the app and getting immediate feedback as well as being measured every study visit. Most participants felt that they needed to be held accountable to increase their daily steps. As 1 person noted:

As time goes on you tend to get on to other things, so having something that I am kind of accountable for, makes a difference to me. I work better under accountability, just kind of keeping on track. If I was doing this myself, I could easily get lazy.

ID 1115, plus, age 57 years

Several participants from both groups indicated that the reason they signed up for the study was the *accountability factor*, which they felt they needed to *get motivated* and *get moving*:

I loved being in the study. I really did. You motivated me to walk more. But also, I really loved knowing that somebody had my back. You know. That I was accountable to somebody for my walking. And that makes a big difference for me. It was hard for me letting go of that pedometer. I almost cried. But I really miss it.

ID 1165, control, age 59 years

Engaging in the study also generated a sense of responsibility to do their best to achieve good results:

The study provided a way for checks and balances. You had to be true to yourself because you were trying to do well for the study. I wanted the study to be successful and I wanted to fulfill my agreement to do it to the best of my ability. So sometimes I could be out there walking from 8-9 pm because I haven’t finished my steps. So, I felt a responsibility about it. I liked it. I could be in the study forever and I’d be happy.

ID 1091, plus, age 68 years

Accountability could be internal, being accountable to one’s self, or external, being accountable to others, such as the research team, friends, or family. For some, having an accurate pedometer was sufficient to *take accountability of your own life*. As 1 person stated:

The accountability. You know, you wear the pedometer every day and it’s going to tell the truth regardless and so it makes you want to do better, you know, walk more and perform better, even if it’s just a number.

ID 1162, regular, age 51 years

Others, however, seemed to need more external accountability, knowing that they had to report their steps to someone else held them accountable:

The external reinforcement—I liked having the pedometer and having external motivation. I liked being embarrassed if I had to tell you I hadn’t done anything that day.

ID 1053, plus, age 66 years

Having to *check-in* with someone was a critical aspect of external accountability.

### Resources and Environment

In talking about what supports or inhibits their continued physical activity, the environment and resources emerged as an underlying issue. In this theme, we saw a clear difference between the control and intervention groups. Control group participants discussed the fact that they did not receive the same resources as the intervention group, and they were not happy about it:

I felt that I was in the control group and I sort of wished that I had more assistance with exercise information, you know. Information not just support, umm, emotional or psychological support, but yeah support in terms of information.

ID 1224, control, age 59 years

Intervention participants discussed the resources provided by the study in the form of opportunities for walking or exercise groups and information about other forms of exercise. Indeed, some participants bemoaned the loss of this information although it was available if one searched various websites. Participants liked the convenience of a 1-stop resource repository. This resource was important for participants’ ability to find or create walking groups or buddies to enhance their accountability and sustain physical activity.

Embedded in their discussion of resources were barriers to maintain physical activity poststudy. Consistent with the quantitative data, the 2 main barriers addressed were money and time. Although most agreed that having a pedometer helps in tracking and accountability, not everyone bought one after returning the study pedometer at the end of 9 months.

Money also played a significant role in acquiring a pedometer poststudy, as 1 control participant noted:

I really wanted to buy a new pedometer, but I didn’t have money to do it.

ID 1165, control, age 59 years

Although some participants had sufficient income to join a gym or find a personal trainer, most did not, opting to continue walking on their own.

The biggest resource that was lacking was time, and this perspective is consistent with the 12-month survey where lack of time was one of the top 3 reasons for not engaging in physical activity. Although committing time to actively engage in the study was a responsibility participants embraced, once the study was complete, finding time to be active became an issue. The interaction between time and money was addressed as a major problem:

I think that time is still my number one enemy in this regard. Time and money—because we have a great gym nearby, but I can’t really afford a membership. But being able to go somewhere for ½ an hour, in a safe space, as opposed to walking outside at night, would definitely improve my activity levels.

ID 1253, regular, age 34 years

The environment was positively addressed by both groups when talking about the beauty of the physical environment and how it enhanced their walking experience:

I often used my walk to look around, and enjoy looking at people’s gardens, or enjoy the architecture, or the scenery, or whatever is out there, looking for things.

ID 1165, control, age 59 years

Although a few spoke of issues of safety within the environment, especially walking at night, the physical environment was not as critical to their continued engagement in physical activity. Although resources and environment underpin the themes of tracking, technology versus personal touch, and accountability, they also frame discussions about what motivates engagement in physical activity.

### Motivation

Motivation or being motivated was mentioned frequently across both control and intervention groups. Finding motivation was seen as critical to initiate physically active and important to keep one engaged once the study was complete, even if the motivation process was elusive:

So, just find whatever can motivate you, [use] tools or programs that kind of keep track of that. Because it so easy to just fall back and say “Oh, I’m just going to watch TV.”

ID 1115, plus, age 57 years

Tracking, technology, and accountability play important roles in motivation. Control participants highlighted the importance of wearing a pedometer in and of itself in their motivation to be active as it allowed self-tracking and self-accountability. For the intervention group, using pedometers and apps to track, encourage, and assess progress increased their motivation. Participants in both groups commented that the tracking provided by the pedometer and accountability to the study team was important to maintaining their activity.

Conversely, others talked about what either did not motivate them or what reduced motivation once the study tools were returned at 9 months. Unexpectedly, 1 person noted that the technology used in the study was not a motivator:

I was not motivated by the pedometer. You know because that’s the primary reason why I wanted to participate [in the study]. I felt that wearing the pedometer and looking at the number every day, it would be [a] motivating factor for me to make the number increase and it was not.

ID 1127, control, age 41 years

However, others remarked that the loss of the pedometer and the external accountability had a negative impact on their motivation poststudy:

I think giving up the pedometer and knowing that I never had to come back and be measured and everything. I think that just sort of took away my motivation.

ID 1078, regular, age 68 years

Tracking, via technology and personal touch, and accountability were more critical to sustaining physical activity than motivation.

For some, getting a pedometer and tracking themselves seemed to be enough to keep them active. However, others really spoke about the need to create some form of external accountability and encouragement, a *force of accountability*. One of the most common advice for sustaining a walking regime was finding an *activity buddy*, such as a friend, a neighbor, a coworker, a family member, or an activity-focused group. For some, this meant having someone to walk with:

Get a group of people together that are of the same mindset that are going to actually show up and participate, not by yourself. I think the hardest part of being active is doing it alone.

ID 1182, control, age 39 years

Although others preferred to walk alone, they liked the idea of being accountable to someone else:

If you can’t have the mPED study then I think the idea of having a friend or someone that you are accountable to is good. You don’t necessarily have to do it together.

ID 1091, plus, age 68 years

In general, the ultimate goal of physical activity programs is to establish a routine or habit of physical activity. Although habit formation was not necessarily achieved by participants, it was discussed by some participants.

### Habit Formation

Participants from both control and intervention groups discussed the goal of establishing a routine or habit, but more participants from the intervention group brought up this idea than control participants. One control participant talked about wanting to establish a habit but found it difficult:

I learned that I guess that it’s pretty easy to make the habit, but I still have problems of actually making the habit. So, I don’t know, I don’t know, you know you learn.

ID 1268, control, age 62 years

Conversely another participant discussed how the tracking, technology, and accountability of the study helped her establish a habit or routine:

I want to be a little more active, so that’s what motivated me to join the study and I wanted [it] to help me establish a habit of moving. So, since I had to report daily or they are tracking me daily, so it helps me to establish a routine.

ID 1129, control, age 52 years

Intervention participants situated their discussion about habit formation differently, demonstrating their synthesis of what they learned from the study about making changes and creating habits. One woman stated that she learned that developing a habit is difficult:

Umm, that it takes a lot to change a habit. ...it took being conscious many times each day to actually become eventually conscious enough to just do it to have it become part of my routine instead of a big ordeal.

ID 1072, plus, age 58 years

One woman talked about how making *small habitual changes* in activity can add up to more steps per day, whereas another woman addressed the importance of having a measuring tool in habit formation:

The importance of forming a habit and for me the necessity of having a measuring device to motivate me to do it.

ID 1187, regular, age 62 years

However, another woman who stated she had developed a habit of physical activity and no longer felt the need for a measuring tool:

I already formed that habit and I know, uhh, now I use, I don’t use steps. I use time. How much time I walked, so I know how many steps I might have walked, and I use time to increase my uhh, my physical activity.

ID 1289, plus, age 56 years

Another lesson learned was that developing a habit of physical activity does not necessarily mean having to do it every day:

What the study [showed me is] that, umm exercise habits can be developed. Part of my biggest thing before was that I don’t think I’m able to do it, maintain some type of program or routine every day; and the study has totally changed that idea for me and at least I am going to maintain my walking. Probably not as rigorous as it should be but at least it’s a big improvement for myself.

ID 1151, regular, age 42 years

Finally, several women discussed the importance of having social support, through family and friends, to motivate them to develop a physical activity habit:

Anything that can kind of motivate you. I got my sister on that walking telephone thing. She went out and bought a pedometer. And she way passed me. She is all over it now. Prior to that she didn’t know what she was doing. And then she got the pedometer and it was like Okay. ... So umm, so just find whatever can motivated you, tools or programs that kind of keep track of that.

ID 1115, plus, age 57 years

## Discussion

### Principal Findings

Feedback from women’s experiences and perspectives of physical activity after completing the mPED trial reflect complex interactions among internal and external factors. Although we previously reported on the efficacy of the 3-month and additional 6-month effects of a mobile phone app in conjunction with brief in-person counseling on physical activity [22], participants at the 12-month interview spoke about the challenges of maintaining physical activity after the intervention technologies were removed [14,21]. In the 12-month survey, one of the interesting findings was that a significantly higher proportion of participants in the intervention group, compared with the control group, reported engaging in less physical activity from 9 to 12 months. For the intervention group only, the major reason for being less active was that the study had ended. Yet, approximately half of the participants in the intervention group reported that they still regularly wore a pedometer or activity tracker and engaged in brisk walking after the trial ended, and this proportion was higher than in the control group.

These quantitative differences between the intervention and control groups from the 12-month survey results were supported by the findings of the qualitative interviews. The themes identified in this study as influencing women’s continued engagement in physical activity were tracking, technology versus personal touch, accountability, resources and environment, motivation, and habit formation. Although motivation, resources, and accountability have been mentioned in other studies [14,18,20], it is the interaction among all the themes that emerged as particularly important. Researchers have suggested that the factors leading to the adoption of physical activity in the short term are different from those needed to sustain physical activity over time [32,33]. Study participants in this trial seemed to agree that what motivated them to start a physical activity program did not necessarily keep them active after completion of the study. The Physical Activity Maintenance Model [33] suggests that goal setting, motivation, and self-efficacy, mediated by life stress and environment, influence physical activity maintenance. The qualitative data support motivation as an important element in physical activity maintenance and life stress in the form of lack of time and money and environmental resources as mediators of maintaining physical activity. However, self-efficacy did not emerge as a key aspect of maintaining physical activity postintervention. Although participants mentioned the importance of meeting goals, goal setting did not emerge from the interviews. As goal setting was a part of the intervention, participants may not have seen the need to specifically address it. Instead, tracking, both digital and human, and internal and external accountability are what kept participants engaged in physical activity over time.

The trial used the SCM as a framework for the study [28] and posited that the intervention would take people in the contemplation or preparation stages and move them into the action and maintenance stages. Given the fact that all participants in the intervention and control groups increased their physical activity during the trial [22], it is clear that the participants did move into the action stage. Although there was a slight reduction in steps at the end of the maintenance intervention, all the participants continued to engage in higher levels of physical activity compared with their baseline levels. However, reaching the termination stage where physical activity becomes a habit was more elusive, reflecting issues with resources and environment, motivation, and accountability. Both the 12-month survey and qualitative interview findings highlighted the difficulties of habit formation, which is the process by which regular physical activity becomes automatic and routine. However, some participants in the intervention group clearly applied some of the things they had learned during the first 3 months of the trial to successfully develop a physical activity habit or routine.

The findings of this study are also somewhat inconsistent with self-determination theory (SDT) [34] often used by studies investigating exercise motivation. A systematic review of exercise, physical activity, and SDT demonstrated that intrinsic motivation, defined as doing an activity because of the satisfaction it brings, is more predictive of long-term exercise adherence than extrinsic motivation, which is predicated upon a desire for social reward to avoid disapproval [34]. Consistent with the literature, being motivated or finding your motivation was seen as an important aspect of long-term sustainability of physical activity [14,20]. Motivation was seen as an individual resource or need and that each person had to find what motivated them to continue engaging in physical activity. However, motivation was not central to their discussion of what kept them active poststudy. The interaction between tracking, technology versus personal touch, resources and environment, and accountability emerged as more critical to forming their physical activity habits over time.

In both groups, pedometers or activity trackers clearly provided a mechanism for keeping track of one’s steps, providing for self-monitoring of ones’ own physical activity [35]. Being able to see how well they did over the course of the day or week and knowing whether they had reached their goal was helpful to those who bought and wore activity trackers. As such, tracking and technology interact to both encourage and measure physical activity. Indeed, this is what these technologies are designed for, namely promoting self-monitoring of physical activity. However, what was also evident in the data was the importance of the personal touch in tracking. Participants remarked on the importance of knowing that someone on the research team was keeping track of their steps, a form of external monitoring, reflecting a desire to meet research team members’ expectations. Participants noted that they really missed having to report their steps to a study personnel and knowing that they could reach out and talk to a person as needed. This perceived value of both internal (supported by technology) and external tracking (supported through personal touch) is related to participants’ feedback on the importance of accountability.

Being held accountable or the need for accountability was mentioned frequently by participants and therefore emerged as critical to both motivation and physical activity habit formation. Those who remarked on the value of physical activity trackers and the importance of internal motivation also indicated that one had to be self-accountable. Use of tracking technology provided the means to determine if one had met one’s goals, thereby keeping oneself accountable. However, many of the participants struggled with lack of accountability at the end of the 9-month maintenance intervention, regardless of whether they owned a pedometer or not. The removal of the intervention technology, while problematic for some, was less of an issue than the absence of external tracking by research staff. Many participants remarked that not having the external monitoring made them less accountable and took away motivation. Participants also sought out external trackers in the form of activity buddies or walking groups or even family and friends to remain accountable. This need to be accountable to others is consistent with other research exploring exercise adherence in older adults [19,21,36]. Although internal and external accountability are not synonymous with intrinsic or extrinsic motivation, participants in this study revealed that contrary to SDT studies [34], external accountability was necessary for continued engagement in physical activity.

Underpinning the participants’ discussions about sustaining physical activity was the importance of access to and use of resources within their social, economic, and physical environment. Limited economic resources impacted some participants who seemed to struggle a bit more with sustaining their physical activity. Although the participants were encouraged to purchase a pedometer (if they did not have one), budgetary constraints impacted some participants’ ability to purchase a pedometer, whereas others did not perceive the need to buy one. Research has shown that walkability, often measured using built-in environment features such as sidewalks and street connectivity, predicts walking patterns and physical activity [37-39]. Although we did not ask about the physical environment, we expected this to emerge as a reason for not walking. However, participants remarked that the wealth of environmental walking resources available encouraged them to walk and gave them a chance to see the beauty of their neighborhoods.

The resource that was most important and seemed in shortest supply was time. This finding emerged from both the 12-month survey and the qualitative interview and is consistent with research that shows that time scarcity reduces physical activity [40]. Some participants noted that you had to make time or find time to walk or exercise, and most indicated that they committed the time for the sake of the study; but once the study ended, their busy lives took over, and walking or activity time was no longer a priority. The intersection of lack of time and money was also addressed by some of the participants. The need to make money superseded finding time to walk, and both time and money have been addressed in previous research on adherence to exercise among middle-aged women [14,21,40]. Participants generally found the time to engage in physical activity to fulfill their commitment to the study, but this was not enough to keep them fully engaged poststudy.

### Strengths and Limitations

One of the strengths of the study was an excellent retention rate at 12 months. Overall, 97% (203/210) of the randomized participants in the mPED trial were interviewed and analyzed in this qualitative study, enhancing the credibility of the findings. Although interviews lasted, on average, only 18 min, sufficient data were generated to saturate the themes. All interview transcripts were quality checked for accuracy, and the dual coding of 15% (30/203) of the transcripts to achieve 90% agreement enhanced dependability. The qualitative analysis was supported by the quantitative findings from the survey, enhancing confirmability [41,42].

Despite these strengths, there are limitations to this study. As the digital technologies used by the trial to measure physical activity in the form of steps per day had been removed from the participants at 9-months, we relied on participants’ self-perception of their physical activity postintervention, which is subject to bias. Transferability of the findings of this study may be limited because of the unique social and environmental factors present in the San Francisco Bay Area. In addition, the study participants were women; therefore, these findings may be applicable to men and children.

### Conclusions and Implications

Both the 12-month survey and qualitative interview findings highlight the experiences and perspectives of physically inactive women who participated in the mPED trial. As the 12-month survey did not objectively measure participants steps for the last 3 months of the study, we cannot determine the maintenance of physical activity at 12 months. However, a higher proportion of the participants in the intervention group reported regular wearing of the pedometer and more brisk walking than the control group, indicating continued engagement in physical activity. Tracking via technology and personal touch and accountability emerged as central factors in initiating and maintaining activity over time. Resources, in the form of time and money, supported or impeded continued engagement in physical activity and habit formation. Digital technology, especially in the form of activity trackers, is moving faster than research, providing more opportunities to harness this dynamic interactive process in promoting and forming physical activity habits among inactive women.

## Appendix

Multimedia Appendix 1 Overall study design and interview questions.

Multimedia Appendix 2 Comparison of thematic quotes between control and intervention groups.

## Abbreviations

mPED: mobile phone–based physical activity education
RCT: randomized controlled trial
SCM: Stages of Change Model
SDT: self-determination theory

## References

[1] Troiano RP, Berrigan D, Dodd KW, Mâsse LC, Tilert T, McDowell M (2008). Physical activity in the United States measured by accelerometer. Med Sci Sports Exerc.

[2] Tucker J, Welk G, Beyler NP (2011). Physical activity in US: adults compliance with the Physical Activity Guidelines for Americans. Am J Prev Med.

[3] Rossi A, Dikareva A, Bacon SL, Daskalopoulou SS (2012). The impact of physical activity on mortality in patients with high blood pressure: a systematic review. J Hypertens.

[4] Ford E, Li C, Zhao G, Pearson W, Tsai J, Churilla JR (2010). Sedentary behavior, physical activity, and concentrations of insulin among US adults. Metabolism.

[5] Gallaway P, Miyake H, Buchowski M, Shimada M, Yoshitake Y, Kim A, Hongu N (2017). Physical activity: a viable way to reduce the risks of mild cognitive impairment, alzheimer's disease, and vascular dementia in older adults. Brain Sci.

[6] Gonçalves AK, Florencio GL, Silva MJ, Cobucci RN, Giraldo PC, Cote NM (2014). Effects of physical activity on breast cancer prevention: a systematic review. J Phys Act Health.

[7] Mammen G, Faulkner G (2013). Physical activity and the prevention of depression: a systematic review of prospective studies. Am J Prev Med.

[8] Olver IN (2016). Prevention of breast cancer. Med J Aust.

[9] (2018). Health.

[10] Winston G, Phillips E, Wethington E, Devine C, Wells M, Peterson J, Hippolyte J, Ramos R, Martinez G, Eldridge J, Charlson M (2015). Social network characteristics associated with weight loss among black and hispanic adults. Obesity (Silver Spring).

[11] Ward BW, Nugent CN, Schiller JS Centers for Disease Control and Prevention.

[12] Pew Research Center.

[13] Krebs P, Duncan DT (2015). Health app use among US mobile phone owners: a national survey. JMIR Mhealth Uhealth.

[14] Crain AL, Martinson BC, Sherwood NE, O'Connor PJ (2010). The long and winding road to physical activity maintenance. Am J Health Behav.

[15] Fukuoka Y, Gay C, Haskell W, Arai S, Vittinghoff E (2015). Identifying factors associated with dropout during prerandomization run-in period from an mHealth physical activity education study: the mPED trial. JMIR Mhealth Uhealth.

[16] Fukuoka Y, Vittinghoff E, Hooper J (2018). A weight loss intervention using a commercial mobile application in Latino Americans-Adelgaza trial. Transl Behav Med.

[17] Fukuoka Y, Vittinghoff E, Jong S, Haskell W (2010). Innovation to motivation--pilot study of a mobile phone intervention to increase physical activity among sedentary women. Prev Med.

[18] Fjeldsoe B, Neuhaus M, Winkler E, Eakin E (2011). Systematic review of maintenance of behavior change following physical activity and dietary interventions. Health Psychol.

[19] Floegel T, Giacobbi PR, Dzierzewski J, Aiken-Morgan A, Roberts B, McCrae C, Marsiske M, Buman MP (2015). Intervention markers of physical activity maintenance in older adults. Am J Health Behav.

[20] Lefler L, Jones S, Harris B (2018). Key strategies for physical activity interventions among older women: process evaluation of a clinical trial. Am J Health Promot.

[21] McArthur D, Dumas A, Woodend K, Beach S, Stacey D (2014). Factors influencing adherence to regular exercise in middle-aged women: a qualitative study to inform clinical practice. BMC Womens Health.

[22] Fukuoka Y, Haskell W, Lin F, Vittinghoff E (2019). Short- and long-term effects of a mobile phone app in conjunction with brief in-person counseling on physical activity among physically inactive women: the mPED randomized clinical trial. JAMA Netw Open.

[23] Sandelowski M (2000). Whatever happened to qualitative description?. Res Nurs Health.

[24] Sandelowski Margarete (2010). What's in a name? Qualitative description revisited. Res Nurs Health.

[25] Fukuoka Y, Haskell W, Vittinghoff E (2016). New insights into discrepancies between self-reported and accelerometer-measured moderate to vigorous physical activity among women - the mPED trial. BMC Public Health.

[26] Fukuoka Y, Komatsu J, Suarez L, Vittinghoff E, Haskell W, Noorishad T, Pham K (2011). The mPED randomized controlled clinical trial: applying mobile persuasive technologies to increase physical activity in sedentary women protocol. BMC Public Health.

[27] Bandura A, Adams N, Hardy A, Howells Gn (1980). Tests of the generality of self-efficacy theory. Cogn Ther Res.

[28] Prochaska J, DiClemente CC (1983). Stages and processes of self-change of smoking: toward an integrative model of change. J Consult Clin Psychol.

[29] Rubin H, Rubin I (2012). Qualitative Interviewing: The Art of Hearing Data.

[30] Creswell JW, Poth CN (2018). Qualitative Inquiry And Research Design: Choosing Among Five Approaches. Fourth Edition.

[31] Cutcliffe J, Harder HG (2009). The perpetual search for parsimony: enhancing the epistemological and practical utility of qualitative research findings. Int J Nurs Stud.

[32] Blue S (2017). Maintaining physical exercise as a matter of synchronising practices: experiences and observations from training in Mixed Martial Arts. Health Place.

[33] Nigg C, Borrelli B, Maddock J, Dishman RK (2008). A theory of physical activity maintenance. Appl Psychol-Int Rev.

[34] Teixeira P, Carraça EV, Markland D, Silva M, Ryan RM (2012). Exercise, physical activity, and self-determination theory: a systematic review. Int J Behav Nutr Phys Act.

[35] Finkelstein E, Haaland B, Bilger M, Sahasranaman A, Sloan R, Nang E, Evenson Kr (2016). Effectiveness of activity trackers with and without incentives to increase physical activity (TRIPPA): a randomised controlled trial. Lancet Diabetes Endo.

[36] Osuka Y, Jung S, Kim T, Okubo Y, Kim E, Tanaka K (2017). Does attending an exercise class with a spouse improve long-term exercise adherence among people aged 65 years and older: a 6-month prospective follow-up study. BMC Geriatr.

[37] Koohsari MJ, Owen N, Cerin E, Giles-Corti B, Sugiyama T (2016). Walkability and walking for transport: characterizing the built environment using space syntax. Int J Behav Nutr Phys Act.

[38] (2013). National Academies.

[39] Owen N, Cerin E, Leslie E, duToit L, Coffee N, Frank L, Bauman A, Hugo G, Saelens B, Sallis JF (2007). Neighborhood walkability and the walking behavior of Australian adults. Am J Prev Med.

[40] Venn D, Strazdins L (2017). Your money or your time? How both types of scarcity matter to physical activity and healthy eating. Soc Sci Med.

[41] Fukuoka Y, Lindgren TG, Mintz YD, Hooper J, Aswani A (2018). Applying natural language processing to understand motivational profiles for maintaining physical activity after a mobile app and accelerometer-based intervention: the mPED randomized controlled trial. JMIR Mhealth Uhealth.

[42] Holloway I, Wheeler S (2010). Qualitative Research in Nursing and Healthcare. Third Edition.

