# Supplementary material for: Perceptions and Experiences of Women Participating in a Digital Technology–Based Physical Activity Intervention (the mPED Trial): Qualitative Study
Source: JMIR Public Health Surveill. 2019 Dec 20;5(4):e13570. doi: 10.2196/13570 (PMC6942195; doi:10.2196/13570)
Supplement: Multimedia Appendix 1 [file publichealth_v5i4e13570_app1.pdf]

Appendix A

Overall study design

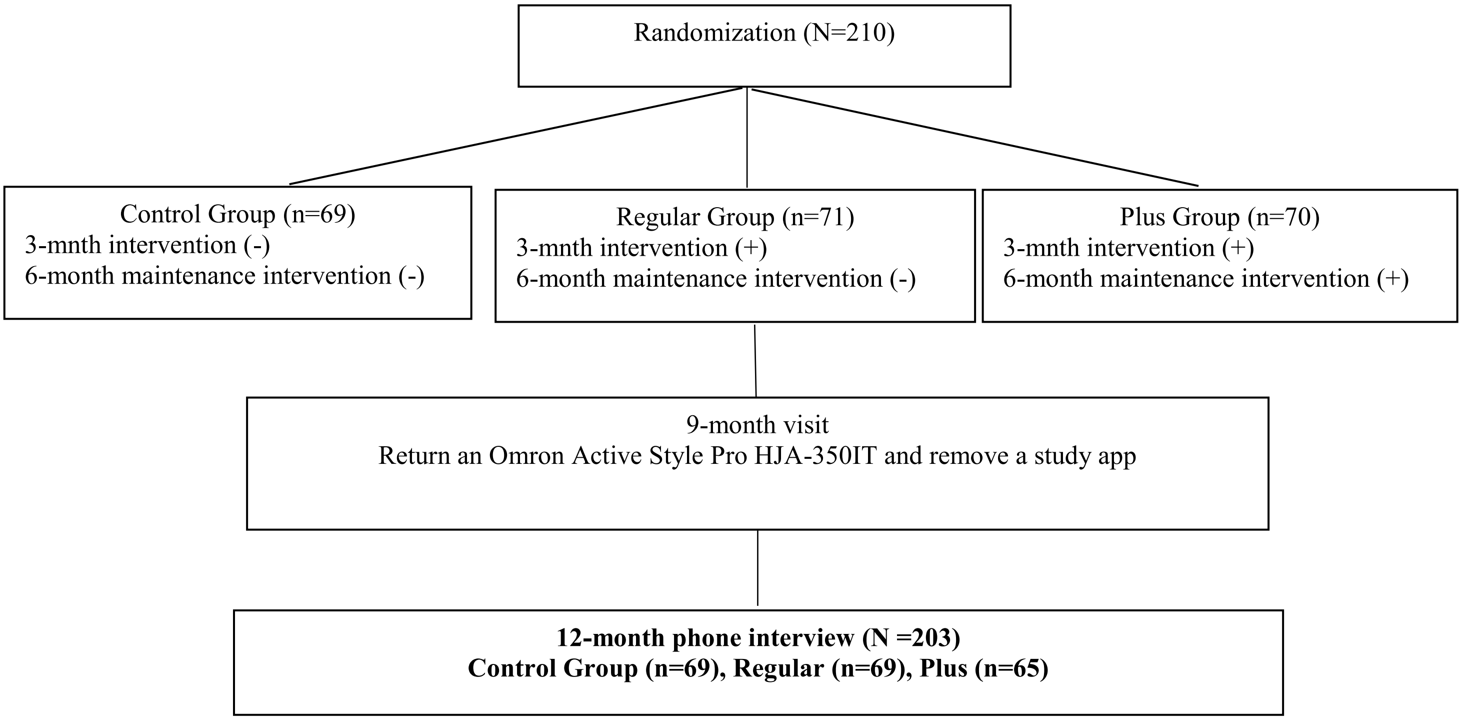

Appendix B

<u>mPED Trial : 12 Month Telephone Interview Questions</u>

Congratulations for completing 12 months of the mPED Study! I would like to take a few minutes to ask some questions and get your honest feedback about our study. We greatly appreciate all of your help with the study and welcome any feedback you may have. This portion of the visit will be recorded.

Think back over the past 12 months of the mPED study to answer the following questions. There is no right or wrong answer. Please be as honest as possible when answering these questions.

1. Looking back over the last 12 months, what did you learn the most from the mPED Study?

2. What did you like the most about the study?

3. What did you like the least about the study?

4. What would you change about the study?

5. Since you returned your study equipment at your 9 month visit, what do you feel would have motivated you more to maintain your physical activity?

6. What advice would you give to other women to help increase their physical activity?

7. Is there anything else you would like to add?

Thank you for your participation! If you know anyone that would be interested in joining our study, please let us know.
