# Supplementary material for: Perceptions and Experiences of Women Participating in a Digital Technology–Based Physical Activity Intervention (the mPED Trial): Qualitative Study
Source: JMIR Public Health Surveill. 2019 Dec 20;5(4):e13570. doi: 10.2196/13570 (PMC6942195; doi:10.2196/13570)
Supplement: Multimedia Appendix 2 [file publichealth_v5i4e13570_app2.docx]

Multimedia Appendix 2

Comparison of thematic quotes between the control and intervention groups.

| Themes | Control group quotes (study ID, group, age of participant) | Intervention (regular and plus) group quotes (study ID, group, age of participant) |
| --- | --- | --- |
| Tracking | “I once thought of myself as a walker, but I guess wasn’t walking as much as I thought I was. Once we started actually counting the steps, I realized “Oh I am not getting in as much as I should” and so the pedometer helped me keep track and put in those extra steps.” [ID 1165, control, age 59 years]; “That I got to wear a pedometer and that I found that I was sitting around a lot. And you know knowing that I am not moving around is thing that now am conscious of.” [ID 1063, control, age 65 years]; “Having an active daily record because the leader in me then went back and measured the steps per day. Because I used it every day and not specifically was going for a walk. Like I would put the pedometer on and say, ‘oh okay now I am going to go for a walk’ and turn on the Nike running. But that was because it was always on.” [ID 1108, control, age 63 years]; “You think you’re doing really well and when it is actually quantified by somebody else you realize ‘Ooh, maybe I am not doing as much as I thought.’” [ID 1165, control, age 59 years] | “I think since the first part of it, I did have the app, I think that really raised my awareness to be able to track, And that is what motivated me to get a activity tracker. Because I did like that feedback.” [ID 1181, regular, age 39 years]; “How easy it is to really do 10000 steps a day once you get motivated to do it and that they’re different mechanisms to keep track of that as well as it was an awareness of areas and places to go to be out more and to enjoy the exercise, the walking.” [ID 1002, regular, age 58 years]; “I learned having a pedometer could be a good spur to tracking how much exercise you are getting and therefore getting more.” [ID 1008, plus, age 58 years]; “Having a goal every day, walking every day, but hated it sometimes. A couple of times I did 20,000 because I was at tradeshows. I think just having a metric to look at every single day.” [ID 1015, regular, age 63 years] |
| Technology versus personal touch | “I liked having the pedometer. Being able to see even the little bit of activity, like oh walking to the printer or walking across the street to grab lunch, it all adds up. Just reporting on myself. That somebody was actually asking, what did you do...the inquiry. Without guilt I should add. I never felt guilty for my inactivity.” [ID 1182, control, age 39 years]; “I think that it was always nice to come there for that reason because you guys were always very pleasant and easy to work with. And you know I loved the reminders that were sent out. I am really sad when I don’t have the reminders, so I really appreciate those reminders that were allowed for us. And I think that that was great.” [ID 1238, control, age 57 years] | “I liked wearing the pedometer the most. I was definitely hesitant at the beginning because it seemed so big, but having that daily feedback and monitoring was incredibly helpful. And I intend to always wear one.” [ID 1175, plus, age 27 years]; “I had three months before where I wasn’t coming in, right? But I knew that, you know, I better have my steps in because I would be called to account for my lack of steps. Not really, but it would be you looking over my shoulder and that would be—I like that.” [ID 1259, regular, age 64 years]; “It’s funny but initially I think I kind of resented it but the monitoring, the quarterly interviews, the talks, initially it was kind of annoying me, and even when you emailed me recently I was like ‘not that again’ but it was actually very helpful.” [ID 1055, plus, age 57 years]; “I’m not a mobile phone user. I think I would have done better with an actual support group dealing with real people other than technology. If there had been a way of me taking responsibility for the recording and tracking-that would have been powerful as well. If I could generate a weekly report or monthly report for myself.” [ID 1008, plus, age 58 years]; “That it gave me that outside monitoring or push that I needed to continue...Yeah, I have to report to it, and somebody is looking at it.” [ID 1151, regular, age 42 years] |
| Accountability | “I liked the accountability of the pedometer and the switching from just the pedometer to one with the application, I liked the application. I think it’s nice that you can kind of check in there and get some sort of feedback. I liked that.” [ID 1166, control, age 60 years]; “I would tell them to get a companion, some kind of exercise or fitness partner. It helps you because of the accountability thing. And I also would say that having a pedometer is helpful as well because you can look at and see what you did over the course of the last few days or something. When I did have the pedometer I would look at it and say “Oh look I got this amount of steps on this day” but then I’ll go, “Oh man I was really like sedentary I only did this amount of steps so let me try to put myself and take more, you know. I think that kind of helps too. And I think having a human being to go with, having a partner or friend or somebody to hold you accountable.” [ID 1272, control, age 38 years] | “I always need to be conscious about moving, about physical activity. If I don’t think about it, I don’t do it. And holding myself accountable—the pedometer and that app really forced me to hold myself accountable.” [ID 1245, regular, age 49 years]; “The accountability. You wear the pedometer every day and it’s going to tell the truth so it makes you want to do better, it makes you want to walk more and perform better, even if it’s just a number.” [ID 1162, regular, age 51 years]; “One of the things that I enjoyed about it was being able to talk with people with whom I’m pretty close with, for instance the people at church. They are checking in on me and asking me how many steps I have done this far, are you walking out. So, it was kind of a communal thing for me. So, besides the fact that I was walking alone, there were people watching to for me.” [ID 1050, plus, age 67 years] |
| Environment, resources, and support | “I like having a pedometer and I actually do kind of miss it even though I haven’t done anything about it yet. I am kind of a number person, so quantifying things is kind of worked the right part of my brain. So even though I haven’t done it, yet I am sure that I will get a pedometer.” [ID 1038, control, age 55 years]; “Find a place to walk that’s really pleasant. I know there are people who play golf and do that, but you do have to enjoy what you are doing...” [ID 1108, control, age 63 years]; “Well I did purchase my own pedometer so I am monitoring my own activity. So I know that there are some days that it is going to be very little, but I kind of forgive myself for that because I know that I will make it up on another day. So I can watch what I am doing and don’t become obsessive about it.” [ID 1009, control, age 63 years]; “I think if, actually if I had purchased one, it would have been a continual reminder. Yeah cause it’s kinda like, you know, I didn’t, I wasn’t accountable. You know where the actual steps were documented.” [ID 1298, control, age 59 years] | “Maybe if there was some kind of group portal where the participations could give each other feedback, suggestions, or encouragement. Because I think it is part of the accountability factor and community factor.” [ID 1181, regular, age 39 years]; “The pedometer. Something to make it easier on me. At that point, I was more motivated to do it. I sort of generally plan on doing it [buying one], but it’s low priority.” [ID 1008, plus, age 58 years]; “The Fitbit—when you recommended and vetted 6 pedometers, that gave me the continuity to continue. I showed 4 other people to buy the Fitbit also.” [ID 1015, regular, age 63 years]; “I used my really demanding work schedule as an excuse maybe, to not be as active as I could be. I think I learned that the level required to maintain a healthy walking schedule is doable if you make it a priority.” [ID 1225, regular, age 57 years] |
| Motivation | “I actually did like the pedometer because it tells me how I am doing that day comparing to how I did previous days and I guess in a way it motivates me to do as much or more than the previous days. It, you know, to keep improving.” [ID 1287, control, age 44 years]; “I think that if I feel that if my status gets tracked on a regular basis, then I feel more motivated to walk because I know that after a few months I will have to answer questions on a questionnaire. So, I feel being tracked provides motivation to continue to do activity.” [ID 1248, control, age 40 years]; “Well, I have more time to exercise now. I have more motivation than I had before because I have more time. I think what would really motivate me is if I saw the results. I am not losing any weight so I don’t see any changes so that would motivate me.” [ID 1230, control, age 28 years] | “I felt a certain amount of pressure, and I mean that in a positive way, gonna be in this study and the reason I did this study was to move more, and if that’s really why I was doing it, I should probably move more and I did find myself thinking that way.” [ID 1284, plus, age 45 years]; “I did feel this psychological accountability to the study when I was part of the study and when I was wearing your pedometer—which was a good thing. In other words, I would wear the pedometer every day and I knew I was part of a study and it was a motivator just knowing I was part of the study. So, what would have motivated me more, would be to still be part of a study, even if I don’t have your pedometer anymore.” [ID 1193, regular, age 60 years]; “The pedometer. Like I said, it really pushed me into exercising more and putting in more steps. I’ve never had any good motivation and I wasn’t really pushing myself if I felt tired, when I need to do the exercise, I just stop. Now with the goal of 10,000 steps, I just put in the steps. So, I have to buy a pedometer after this.” [ID 1194, regular, age 46 years]; “I thought that I’d be more motivated, but it’s easy to find excuses not to do things. I feel I should have done more probably. I know that for sure. It’s just that all the things that came up kids going to school, going here and there, going to college. I think that the pedometer motivates me.” [ID 1318, regular, age 58 years]; “I like coming in and talking with you guys and seeing if I can get my steps up and it was motivational to have somebody to talk to.” [ID 1269, regular, age 59 years]; “Walking really can help, really can motivate you and help you to be physically active. Also, tracking that, tracking and walking.” [ID 1251, plus, age 52 years] |
| Habit formation | “I did purchase my own pedometer, so I am monitoring my own activity. I know that there are some days that it is going to be very little, but I kind of forgive myself for that because I know that I will make it up on another day. So, I can watch what I am doing and don’t become obsessive about it.” [ID 1009, control, age 63 years]; “I think, I do think it can be as simple as walking outside in your own neighborhood. If you start doing laps around your street, laps around your block, do it every day, and then it becomes routine. I would feel that it doesn’t have to necessarily be, you know, a gym membership or a gym class. Just getting into the habit and finding others to go outside with and be with. And a cup of coffee helps when you aren’t doing it.” [ID 1312, control, age 41 years]; “There is something about keeping track because it is very difficult to discipline yourself, the whole things is to get a new habit. So, wearing your pedometer and having to wear it, there was nothing I could do about it. I had to wear it. Because, in the beginning it as like ‘Oh my gosh, I have to wear this every day’ but after a while you see, that this thing will work. You know you just do it.” [ID 1060, control, age 50 years] | “That I could fit in activity, I could just make small habitual changes that could add up.” [ID 1181, regular, age 39 years]; “Exercise habits can be developed. Part of my biggest thing before was that I don’t think I’m able to do it, maintain some type of program or routine everyday and the study has totally changed that idea for me and at least I am going to maintain my walking. Probably not as rigorous as it should be but at least it’s a big improvement for myself.” [ID 1151, regular, age 42 years]; “That I do have time, that I can squeeze time in to do what that need to be done, usually. I was also kind of hoping that after doing it that long, it would become, what do they say, it takes 21 days to make a habit?” [ID 1064, regular, age 65 years]; “Oh yeah, I just wanted to say that I felt really that did this survey or did this program and sort of that it did change my life...in a lot of ways just by, because it was so long, it really did change my habits and it forced me and my husband to go out on walks, to be more active when we use to also say we would do it, but this study kind of forced me to actually do that. So I really appreciate everything that you guys did and I am really glad that I was part of this study.” [ID 1093, regular, age 28 years]; “The importance of forming a habit and for me the importance of having a measuring device to motivate me to do it.” [ID 1187, regular, age 62 years]; “Walking has become a habit. That’s, that’s the best thing I think, so I feel, I feel a little bit guilty if I don’t walk. (Ok. Alright, was there anything else that you liked about the study?) Anything else I liked about the study. I’m starting to invite my friends or families to walk with me, so I’m not just helping myself. I help my friends, as well.” [ID 1289, plus, age 56 years] |
